# Arthroscopic Reduction and Internal Fixation for Peritrapezium Traumatic Axial Carpal Dislocation: A Case Report

**DOI:** 10.7759/cureus.31387

**Published:** 2022-11-11

**Authors:** Shogo Shibata, Koichi Yano, Yasunori Kaneshiro, Takuya Yokoi, Hideki Sakanaka

**Affiliations:** 1 Department of Orthopaedic Surgery, Seikeikai Hospital, Sakai, JPN

**Keywords:** ligament, dislocation, fracture, arthroscopy, trapezium

## Abstract

Axial carpal dislocations and fracture-dislocations are rare injuries involving derangement of the carpal arches. Several surgical approaches have been reported as a means of treatment, including the use of closed or open reduction and internal fixation. However, to our knowledge, surgical treatment using arthroscopy has not been reported so far. Here we present the case of a 54-year-old man who experienced peritrapezium axial carpal dislocation while reversing his car. Minimally invasive surgery using arthroscopy was performed because of severe swelling of the hand. Reduction under arthroscopic assistance using midcarpal portals was performed based on the relationship between the trapezium and trapezoid, and fixed wires were inserted. Wires were removed at six weeks postoperatively, and range-of-motion exercises of the wrist joint were started. At one-year postoperative follow-up, the patient was asymptomatic, with no difficulties while performing daily activities and work. Computer tomography images revealed an anatomical carpal arch without traumatic arthrosis. Arthroscopy-assisted surgery enabled us to anatomically reduce fracture-dislocation of the trapezium and assess the injury path.

## Introduction

Axial carpal dislocations and fracture-dislocations are rare wrist injuries, and they are usually caused by high-energy trauma [1-3]. These injuries involve derangement of the distal carpal row and metacarpal arch and result in flattening of the proximal and distal transverse arches of the wrist. Surgical treatments using closed or open reduction and internal fixation have been described [1,4,5]. However, to our knowledge, no surgical approaches using arthroscopy have been reported so far.

In this case report, we present a minimally invasive arthroscopy-assisted surgical technique, describe the suspected mechanism of injury, and report the clinical outcome of a patient with peritrapezium axial carpal dislocation.

## Case presentation

**Figure 1:**
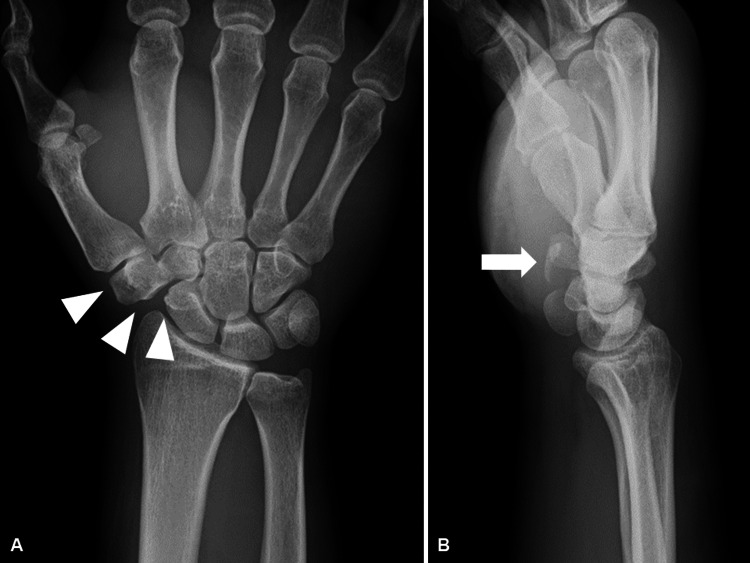
Preoperative plain radiography. (A) Anteroposterior and (B) lateral views of the right wrist (arrowheads show dislocation of the trapezium, and arrow shows volar fragment of the trapezium).

A 54-year-old right-handed man complained of right wrist pain. He had hooked his thumb to the door armrest and remained the door open for reversing his car and his thumb had been pulled by the door that had hit a pillar. The patient was a businessman with no history of trauma to the right wrist. Initial examination revealed severe swelling of the right hand and wrist and tenderness on the scaphotrapeziotrapezoid (STT) joint without a neurovascular disorder. Plain radiographs of the right wrist revealed subluxation of the trapezium compared to the uninjured side (Figures 1A, 1B).

**Figure 2:**
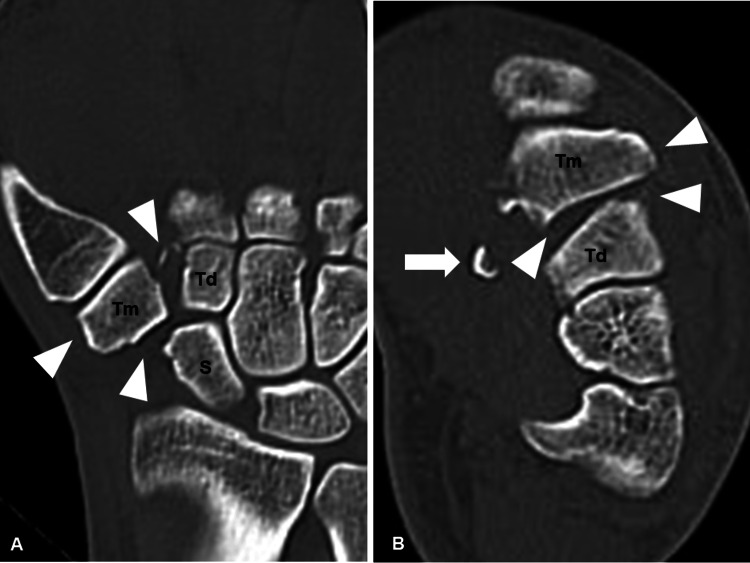
Preoperative plain computed tomography images. (A) Coronal and (B) axial reconstructed computed tomography images (arrowheads show dislocation of the trapezium, and. arrow show volar fragment of the trapezium). S, scaphoid; Td, trapezoid; Tm, trapezium

Plain computed tomography (CT) revealed subluxation, volar fragment of the trapezium, and derangement of the carpal arch (Figures 2A, 2B).

We diagnosed the patient as a case of peritrapezium axial carpal dislocation and decided on minimally invasive arthroscopic treatment.

**Figure 3:**
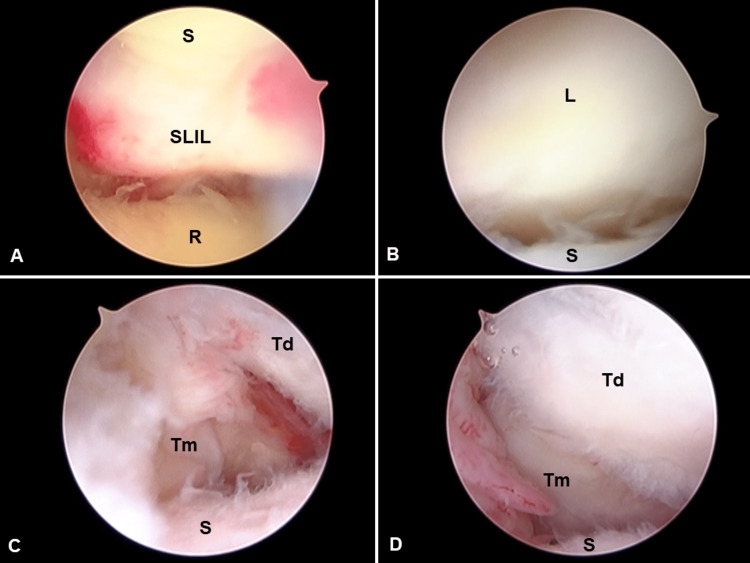
Intraoperative arthroscopic photographs. (A) Radiocarpal joint. (B) Midcarpal joint. (C, D) Scaphotrapeziotrapezoid joint. S, scaphoid; R, radius; SLIL, scapholunate interosseous ligament; L, lunate; Td, trapezoid; Tm, trapezium

Surgical treatment was performed under regional anesthesia four days post-injury. Examination under anesthesia revealed that the thumb was unstable around the STT joint. The dislocation could not be reduced by closed and manual manipulation including traction to distal and pushing from the dorsoradial direction. A 1.9-mm arthroscope (Stryker K. K., Tokyo, Japan) was used under wet conditions. Using a traction tower (Arc Wrist Tower, Acumed LLC, Hillsboro, OR) with a finger trap on the thumb and middle finger at 10 pounds, 3-4 and 6R portals were created, and the radiocarpal joint was observed. The scapholunate interosseous ligament (SLIL) was attenuated with hemorrhage (Figure 3A). The triangular fibrocartilage complex and cartilage were not injured. Midcarpal radial (MCR) and midcarpal ulnar (MCU) portals were created, and the midcarpal joint was observed. The SLIL injury was classified as a grade 3 injury according to the Geissler classification (Figure 3B), while the lunotriquetral interosseous ligament was not injured [6]. A scaphotrapezial portal was created, and the STT joint was observed. It revealed fibrillation of the trapezoid and scaphoid cartilage. A hematoma was observed at the radial side of the trapezoid. After resection of the hematoma and removing of the ruptured dorsal trapeziotrapezoid ligament at the fracture site, which were supposed to obstruct the manual reduction using a shaver, volar fragment of the trapezium was noted (Figure 3C). The trapezium was subluxed dorsally. Traction was loosened until aligned to the joint level between the trapezium and trapezoid. Trapezium was reduced using a finger trap and bone clamp while reducing the gap and dorsal contour between the trapezium and trapezoid (Figure 3D).

**Figure 4:**
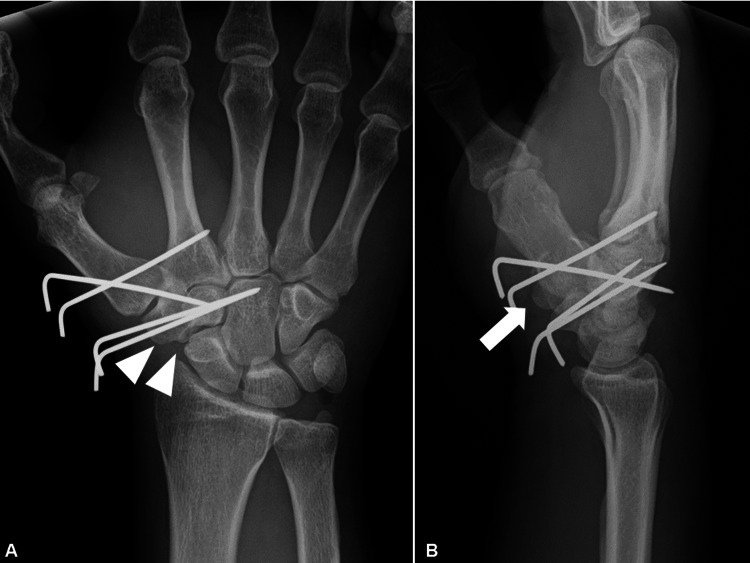
Postoperative plain radiography. (A) Anteroposterior and (B) lateral views of the right wrist (arrowheads show reduction of the trapezium, and arrow shows reduction of the volar fragment of the trapezium).

The trapezium and first metacarpal bone were fixed using Kirchner wires (Figures 4A, 4B), and the wrist was immobilized using a short arm splint.

Finger motion exercises were initiated the day after surgery. The postoperative course was uneventful. At six weeks postoperatively, the wires were removed, and wrist motion exercises were started. Free activity was allowed at three months postoperatively.

**Figure 5:**
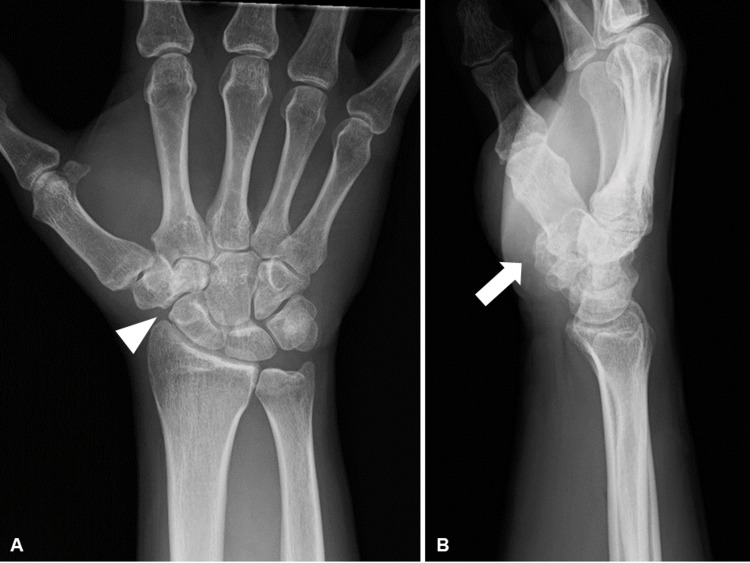
Plain radiography at one year postoperatively. (A) Anteroposterior and (B) lateral views of the right wrist (arrowhead shows joint space of the scaphotrapeziotrapezoid joint, and arrow shows volar fragment of the trapezium).

**Figure 6:**
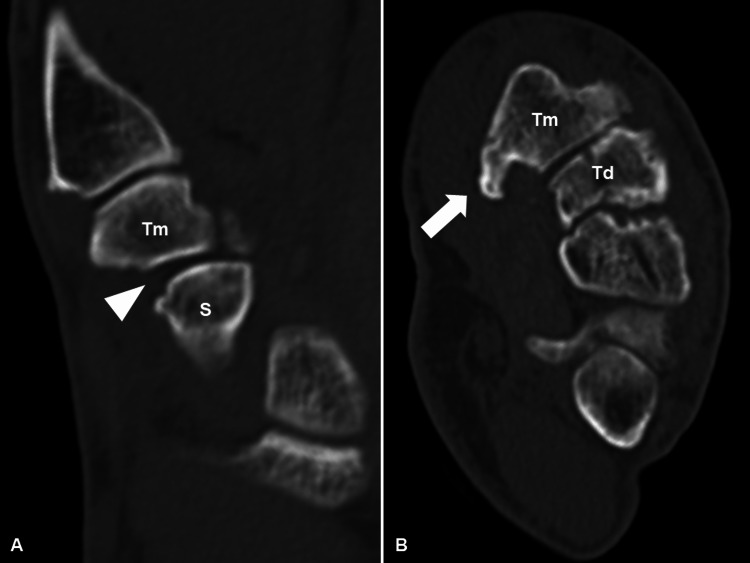
Plain computed tomography at one year postoperatively. (A) Coronal and (B) axial CT images (Arrowhead shows joint space of the scaphotrapeziotrapezoid joint, and arrow shows bony union of the volar fragment of the trapezium). S, scaphoid; Td, trapezoid; Tm, trapezium

One year after the procedure, bone union between the trapezium and volar fragment was observed. Carpal alignment was maintained without traumatic arthrosis and dorsal intercalated segment instability deformity (Figures 5, 6).

The patient’s wrist was asymptomatic during daily activities and work, and the scaphoid shift test was negative. The grip strength of the right and left hands were 44.3 and 44.2 kg, respectively, and the key pinch were 9.8 and 11.4 kg, respectively. The ranges of motion of the right and left upper extremities were as follows: radial abduction of the thumb, 50° and 50°; volar abduction of the thumb, 45° and 45°; wrist flexion, 75° and 75°; wrist extension, 75° and 75°, respectively. The Disability of the Arm, Shoulder, and Hand score was 0.8.

Written informed consent was obtained from the patient for publication of this case report and the accompanying images.

## Discussion

Traumatic axial carpal dislocations and fracture-dislocations are both severe traumas resulting from crush or blast injuries [3]. Most previously reported cases of peritrapezium axial carpal dislocation and trapezium dislocation involved patients sustaining high-energy trauma. The direction of the force in most cases was considered as anteroposterior due to flattening of the carpal arch, and longitudinal disruption of the carpus attached metacarpals. Over 90% of these traumas are open injuries, and they are often accompanied by extensive soft injuries [2]. Because closed injuries, as reported here, show subtle radiographic changes despite considerable swelling, CT images help define the exact pathology of the trauma.

In the present case, arthroscopic-assisted surgery offered a minimally invasive approach for accurate anatomical reduction. Arthroscopic surgery using five portals did not cause postoperative complications including surgical wound trouble. Moreover, the carpal arch could be repaired under accurate reduction between the trapezium and trapezoid. Traumatic arthrosis was not noted, although the postoperative follow-up period of one year was short. The carpal arch is a curved alignment that makes assessing the status of reduction using intraoperative fluoroscopy difficult. Surgical approaches involving closed or open reduction and internal fixation have all been reported to manage similar trauma. In contrast, some reports have shown subluxation of the trapezium and destruction of the carpometacarpal joint on plain radiography [4,5,7-20]. We believe that reduction using arthroscopy enabled us to remove the inhibitors of reduction and to obtain complicated alignment over both the closed reduction and internal fixation and the open reduction and internal fixation. The clinical result was satisfactory, although grip and pinch strength did not recover to the extent of the uninjured side. Concerning to the state of the ligaments, dorsal trapeziotrapezoid ligament was injured. Volar trapeziotrapezoid ligament, which could not be seen by arthroscopy, was supposed not to be injured because the volar fragment of the trapezium still remained at its position.

The injury was caused by pulling force, which was suspected to be a complex force including the abduction and distraction, and showed an unusual pattern compared to previous reports. The suspected mechanism of injury based on intraoperative arthroscopic findings was damage to the radial side of the carpus, trapezium, and scaphoid, leading to disruption between the trapezium and trapezoid and SLIL injury. If more severe axial or shear force had been applied from the path of injury, peritrapezium and periscaphoid disruption might have occurred.

## Conclusions

Our patient suffered peritrapezium axial carpal dislocation after his thumb had been pulled. Plain CT images were useful to diagnose the pathology because plain radiography showed subtle changes. We performed surgery under arthroscopic assistance, and we could obtain an accurate reduction and assess the injury path. One year after surgery, carpal alignment was maintained without traumatic arthrosis. We think that arthroscopic assisted surgery for uncommon fracture-dislocation is useful to reduce anatomically and assess the injury path.
